# Revisiting the Master-Signifier, or, Mandela and Repression

**DOI:** 10.3389/fpsyg.2015.02028

**Published:** 2016-01-19

**Authors:** Derek Hook, Stijn Vanheule

**Affiliations:** ^1^Department of Psychology, Duquesne UniversityPittsburgh, PA, USA; ^2^Department of Psychology, University of PretoriaPretoria, South Africa; ^3^Department of Psychoanalysis and Clinical Consulting, Ghent UniversityGhent, Belgium

**Keywords:** discourse, discourse of the master, Lacanian psychoanalysis, Mandela, master-signifier, psychoanalysis, repression, signifier

## Abstract

The concept of the master-signifier has been subject to a variety of applications in Lacanian forms of political discourse theory and ideology critique. While there is much to be commended in literature of this sort, it often neglects salient issues pertaining to the role of master signifiers in the clinical domain of (individual) psychical economy. The popularity of the concept of the master (or “empty”) signifier in political discourse analysis has thus proved a double-edged sword. On the one hand it demonstrates how crucial psychical processes are performed via the operations of the signifier, extending thus the Lacanian thesis that identification is the outcome of linguistic and symbolic as opposed to merely psychological processes. On the other, the use of the master signifier concept within the political realm to track discursive formations tends to distance the term from the dynamics of the unconscious and operation of repression. Accordingly, this paper revisits the master signifier concept, and does so within the socio-political domain, yet while paying particular attention to the functioning of unconscious processes of fantasy and repression. More specifically, it investigates how Nelson Mandela operates as a master signifier in contemporary South Africa, as a vital means of knitting together diverse elements of post-apartheid society, enabling the fantasy of the post-apartheid nation, and holding at bay a whole series of repressed and negated undercurrents.

## Introduction

There is a good deal of excellent literature that explores the Lacanian notion of the master-signifier from the perspective of political discourse theory and ideology critique (Laclau and Mouffe, 1985; Žižek, 1996; Stavrakakis, 1997, 1999; Laclau, 2007). While much is to be gained from this literature, it often neglects salient issues pertaining to the role of master-signifiers in the clinical domain of individual psychical economy. However, the popularity of the concept of the master (or empty) signifier in political discourse analysis and ideology critique has proved a double-edged sword. On the one hand it demonstrates how crucial psychical processes are performed via the operations of the signifier enabling thus a *de-psychologizing* of such processes of identification. On the other, the use of the master-signifier concept within the political realm to track discursive formations and the functioning of ideology tends to distance the term from application in the realm of subjectivity and as an element in the functioning in the unconscious. In short: the master-signifier seems, all too often, more a concept derived from discourse theory than from clinical psychoanalytic practice.

## “In ways I cannot say”

As way of introducing the concept of the master-signifier, one might imagine the following scenario. You are accosted by a camera crew who ask to film you as you list in a few words what is of greatest significance in your life and why. “What,” the interviewer asks you, “would you be prepared to give your life for?” True enough, not everyone would be reduced to a state of stumbling inarticulacy by such a situation. Many might quite happily offer an initial response (“My children,” “The church,” “My country,” “Science,” “Humanity,” etc.). Then again, even those who are able to summon up an appropriate response will doubtless be dogged by a sense of the inadequacy of their words, by their own inability to fully articulate the reasons for the depth of this libidinal investment. Added to this is the inevitable prospect that the words one uses in such situations will seem hopelessly derivative, abstract and formulaic, devoid of any real personalized significance.

Such a situation would be made even more trying should the interviewer press on and on, interrogating each given belief—be it a deeply-held personal, political or even spiritual commitment—with the hystericizing prompt: “*But why?*,” “*Why do you believe* that?” The unavoidable conclusion to such an unrelenting line of questioning would be a circular—and no doubt exacerbated—retort: “*Because I do!*” Such a retort, like that of the exasperated parent's “*Because I said so!”* is sometimes all that can be offered in order to hold a deluge of questions—and inadequate answers—at bay. This situation calls to mind the age-old poetic dilemma: the difficulty of putting into words the reasons one loves one's partner, one's family, one's country, etc. The impossibility of ever fully answering such a question seems perhaps self-evident: being in love presents us with a self-justifying condition which always exceeds the reasons I might give for being in love. Indeed, the reasons I love someone (or something) can never be wholly rationalized or exhausted by a string of signifiers, partly because such signifiers refer on and on to other signifiers without ever “hitting the real.”

## The insufficiency of the signifier

We are presented here with the apparent inadequacy of the signifier; the problem of slippage from one term of reference—unable in itself to fix a given meaning—to another, and then another, and so on *ad infinitum* in an indefinite process which fails to arrest the slide of deferred meaning. This scenario is nicely invoked by Lacan's (2006) at first curious definition of the signifier: “a signifier is what represents the subject to another signifier” (2006, p. 694). There is no naïve psychological realism here; it is not the case that the signifier can transparently represent the subject as he or she really “is.” It is rather the case that what one “is,” or, more accurately, the signifiers that one uses to portray who one is (“business-like,” “fair-minded,” “decisive,” “dedicated”) are, as in the example of a politician's self-portrayals, related still to other signifiers (“presidential,” “exceptional,” etc.).

“The signifier does not provide a guide to reality,” insists Miller (2016), “but presents myriad relations with other signifiers.” A signifier, moreover, is defined by other signifiers, indeed, “by its relation to the chain of *all* other signifiers.”

[S]ignifiers slide along, metonymy gets away from real objects, language lives on its own terms, existing in dimensions far removed from signifieds, doing far more than simple describing them. This is where the subject exists; these are the laws to which we are subjected (Miller, 2016)

In such situations where more can always be said, our best option is, in effect, to tie a knot in discourse. The self-referring quality of a given oft-stressed signifier has to suffice when no over-arching explanation can be given (“Well that's *Jeffrey* for you…!,” “Boys will be boys…”). This, after all, is what self-referential answers do: they don't as much provide sufficient reasons, as loop back on what has already been said, and elevate one signifier over others (“My *children* mean everything to me,” “For me it boils down to *faith*,” “Evolution does not adhere to the principles of *Christianity*”). The signifier here over-reaches its signified; it exceeds what it literally signifies to perform a different discursive function, that of drawing a line, halting a sequence of inadequate explanations by the imposition of a master-signifier (“We know it is true because *science* tells us so”).

In this way such responses enable a temporary point of fixity; they ground a point of belief and/or authority. Bracher (1994) has this tautological logic in mind when he explains how speakers use certain signifiers—master-signifiers—“as the last word, the bottom line, the term that anchors, explains or justifies the claims or demands contained within the message” (p. 112). Receivers of communication respond to master-signifiers similarly: “whereas other terms and the values and assumptions they bear may be challenged, master-signifiers are simply accepted as having a value or validity that goes without saying” (p. 112). Master-signifiers often appear then as those unarguable aspects of a discursive position, as those self-validating points of attachment to a broader ideological or personal worldview.

## The master-signifier in the field of ideology

Helpful as the foregoing illustration may have been, it begs further elucidation. This can be done in two phases: by examining a series of basic themes in political/sociological uses of the term and then by turning to the more clinical literature. In this way we will provide a basis for understanding the concept both in its societal and subjective domains of application, while also pointing to certain of the tensions arising between these two areas of application.

Within any discursive network or “system of signs” there are certain privileged signifiers, what Lacan (1993) initially referred to “*points de capiton*,” nodal-points, which function to “button down” meaning and ensure the smooth exchange of signifiers. Such signifiers are evident at the level of everyday speech, typically as those oft-repeated or affectively-loaded terms which function to ground a given system of references. These signifiers, paradoxically, assume a disproportionate importance in relation to surrounding signifiers. Any number of examples can be supplied here, from master-signifiers operating at an overtly political and ideological societal level (as in the American context: “9–11,” “Freedom,” “Our troops”), to the more idiosyncratic range of master-signifiers as they operate at the level of individual subjectivity to play an organizing functioning in psychical economy. Such signifiers operate to integrate a disparate array of elements, to fashion effects of legibility out of an otherwise indeterminate (and often distressing) set of discursive elements. In his Seminar III Lacan described the role of the master-signifier in the following terms: “Everything radiates out from and is organized around this signifier. It's the point of convergence that enables everything that happens in this discourse too be situated” (1993, p. 268).

A recent definition of the master-signifier—which serves us both as a summary overview and as a basis for critical comparison—is found in *The Žižek Dictionary*:

[B]ecause signifiers refer only to other signifiers, this produces a seemingly endless chain of references…[T]his seemingly infinite sequence of referral can be fixed or anchored only through the intervention of a…'nodal point'…which ‘quilts’ them, stops their sliding and fixes their meanings…this nodal point…in the series of signifiers is the “master-signifier”—a signifier that, although essentially no different from any other signifier, is situated in such a way that it masters the entire sequence of referral by providing a kind of final…guarantee of meaning. It is able to do this…not because it possesses some special significance…but simply because it is able to halt the process of referral by the empty gesture of referring only to itself. This “reflective” signifier is nothing more than a kind of cul-de-sac in the chain of equivalences….‘beneath’ the alleged unity of the field of meaning, there is only a…self-referential, performative gesture (Gunkel, 2014, pp. 190–191).

This definition, admirable in its succinctness and its ability to synthesis the, is indicative in another way also: no mention is made either of the operation of the unconscious or of the fact that the discursive nodal-point of the master-signifier represents equally a nodal-point *of affect*, a point of passionate investment.

A somewhat different account of how the “empty signifier” functions in the consolidation of groups is presented in Laclau's *On Populist Reason*. Laclau (2004) notes how under certain circumstances a given signifier, without ceasing to be particular in what it signifies, “assumes the representation of an incommensurable totality” (p. 70). This signifier is thus “split between the particularity which it still is and….[a] more universal signification” (p. 70). This operation whereby a particular signifier takes up the role of “incommensurable universal signification” is, for Laclau, that of hegemony. Such a task of hegemonic identity requires an empty signifier in which its “own particularity” comes to attain an “unachievable fullness” (p. 71). In contrast to the foregoing definition, Laclau does bring libidinal investment into the picture. The “fullness” of the master-signifier cannot be directly represented as such; “a hegemonic totalization requires a radical investment…the affective dimension plays a central role here” (p. 71).

## The signifier in relation to object A

**Figure 1:**
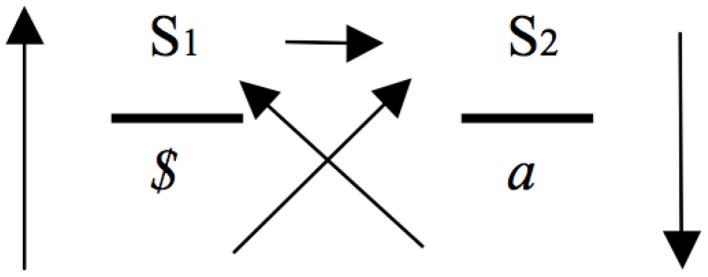
**Discourse of the master (based on: Lacan, 1972, p. 40)**.

One of the reasons why Lacan's work on the signifier cannot be thought of as merely linguistic, is that he takes into account the dimension of the drive, or libidinal investment. This not only means that using the signifier entails libidinous gratification, but above all, that what sets our use of signifiers in motion, is a fundamental deadlock: humans have no automatic harmonious relations with one another, exemplified in the idea *il n'y a pas de rapport sexuel*—there is no such thing as a sexual relationship (Lacan, 1971). That is to say, we need to take into account both the forms of libidinal enjoyment produced in and through speech, and the fact that such linguistic processes are set in motion by an impasse, an impossibility at the level of communication and signification. Indeed, the master signifier only obtains its privileged position in discourse because it comes as an answer to this fundamental deadlock; to the fact that “spontaneous” human relations are marked by an overwhelming *jouissance* we shy away from. In terms of the formula of the discourse of the master (Figure 1), this deadlock is expressed by the absence of an arrow between the truth underlying this discourse (subjective division—*$*) and the product it entails (object *a*, the ever elusive object-cause of desire). The fullness and promise of significance the master-signifier (S1) brings to the fore, is an answer to a fundamental dis-order at the level of the drive.

Interestingly, by pointing out that the discourse of the master produces an object *a*, which further strengthens the use of the master-signifier (expressed by the arrow between S1 and *a*), Lacan posits that inside discourse a libidinous element fuels the use of the master signifier. Our first example of the interviewer and the camera crew made this clear, by stressing the role of a passionate attachment that simultaneously drives and yet defies communicability. In producing (master-)signifiers we behave as if we are seen and listened to. We make sense of what we do and say by acting as if we are actors in ours own lives. We think of ourselves as individuals that might be heard or be seen, and therefore feel the need of stating who we are or what we want. Indeed, the object *a* is the senseless gaze or voice we presume at the level of the other^1^. This element has a disturbing effect, and fuels the production of master-signifiers.

## In the name of Mandela

Collectively, a name starts to function as a master-signifier when, despite the predominance of a general consensual meaning, it comes to signify a great many things to a great many people, all of whom remain in some way identified with—or against—what it is thought to signify^2^. The signifier in question—Margaret Thatcher, Martin Luther King, Chairman Mao, Sigmund Freud, George Washington, Pope John Paul II, Jesus Christ—anchors an array of beliefs and makes a type of (political, religious, ideological) subjectivity possible. In the absence of such a societal master-signifier there is no committed or believing subject, no subject of the group, indeed, no viable group or constituency at all.

### Mandela as….

The name Mandela works in just such a way in post-apartheid South Africa (see for example Barnard, 2014). Evoked by multiple constituencies, Mandela's legacy is pinned to the agendas of divergent interests, and can no longer be assigned any singular meaning. What this means then is that Mandela represents a point of hegemonic convergence in which a variety of incompatible values and identifications overlap. Frederickson's (1990) comment that Mandela succeeded in fulfilling a symbolic role as the “embodiment of the nation that transcends ideology, party, or group” (p. 28) has by now become a historical commonplace. For some Mandela represents the benign, forgiving father of the nation, the embodiment of hope and racial reconciliation; for others Mandela is the radical protagonist of the armed struggle, the revolutionary icon of the African National Congress; for yet others Mandela is a largely de-politicized figure, the commodity image adorning countless accessories and experiences of the “new” South Africa. In terms of Lacan's discourse of the master, this multiplicity is reflected in the upper level of the formula (Figure 1: S1 → S2): master signifiers give rise to various narratives, by means of which meanings arise that might be powerfully divergent.

### …Transcendent signifier

The magic of the master-signifier in the ideological field is that it is able to knit together different constituencies, appealing equally, albeit in very different ways, to a variety of classes who are otherwise opposed in their political agendas. The point of course has frequently been made that Mandela represents fundamentally different things to whites and blacks in South Africa. Nxumalo (2013) for instance comments that “[T]here is a fundamental departure between blacks and whites on what takes precedence in all things that make up this icon called Mandela.” True as this no doubt is, such a contention does not so much oppose as support our argument. Why so? Well, such ideological divergences may ultimately be less important that the fact that whites and blacks find in Mandela a shared reference-point, a common denominator through which their often differing interests may be mediated.

Such a master-signifier enables communication where previously none may have been possible; it represents the possibility that various social antagonisms might (however temporarily) be overcome. It is in this way that a master-signifier makes a type of social bond possible. Historically, this much seems difficult to dispute: Mandela more than any other signifier, proved capable of lending moral purpose and meaning to South Africa's political transition, to the multiple contradictions underlying the post-apartheid era. This is perhaps what proved so anxiety-provoking for many South Africans about Mandela's declining health in late 2013: the prospect that the country would lack a crucial “race mediator,” i.e., a political figure who not only speaks powerfully to black and white groupings, but who also enables them to speak with and to one another.

### …*Empty signifier*

Laclau (2004, 2007) prefers to refer to *empty* as opposed to *master-signifier*s. Doing so draws attention to the fact that master-signifiers have no intrinsic or essential meaning, and that they permit for an endless succession of varying applications and extensions. In the operation of the master-signifier, says Stavrakakis:

a particular signifier is called to incarnate a function beyond its concreteness, it is ‘emptied’ from its particular signification in order to represent fullness in general and to be able to articulate a large number of heterogeneous signifiers…such an empty signifier…serves as a *point de capiton* uniting a whole community (1999, p. 80).

We understand then that master-signifiers can never be totalized or exhausted, that they “sustain the identity of an ideological field beyond all possible variations of positive content” (Žižek, 1989, p. 87). The signifier Mandela, for example, remains ever able to accommodate fresh articulations; it can be appended to a seemingly limitless stream of post-apartheid objects and aspirations. Mandela we might say, is effervescent, an unending signifier, a point of reference for a figure who ascended to the realm of pure symbol. And here the key paradox underlying the concept comes to the fore: the more a master-signifier is heaped with imaginary contents and meanings, the more open and vacuous it becomes.

On the one hand then we have Mandela as an over-arching signifier, the signifier that seemingly encapsulates all that is of value in the post-apartheid context. Yet Mandela is also, a kind of nothingness, a signifier devoid of any absolute or definitive meaning, a signifier that exists at the site of the impossibility of ever saying it all. This much is certainly true: Mandela remains forever indeterminate; one never knows in any final sense what is being invoked when this signifier is being put to use. Mngxitama (2014) points out: “What Mandela, the symbol of freedom, stands for is so vague that anything can be pegged on him.”

### …Positivisation of a void

Of course, as already intimated, the indeterminacy of Mandela is less important than the fact that a system of signification requires a centering-point, a navigational principle, in reference to which all surrounding signifiers gain co-ordinates of meaning and value. Nevertheless, how are we to make sense of the fact that a master-signifier is simultaneously depleted of and yet overflowing with meaning, that—differently put—emptiness and symbolic density here coincide? Simply enough, perhaps, by suggesting that the master-signifier is a signifying operation in which the *nothingness presented by the object* a *is effectively turned into a plenitude of significance*. The impossibility of saying it all is transformed into a surplus.

Glynos (2001) makes a similar point in considering how any complex ensemble of discursive elements—constructions of a nation for example, or of a given society—come to attain a type of relative closure and meaning. Society, for example, lacks an ultimate signifier with which to make it complete:

[N]othing positive can be said about the “truth” of society except that it is incomplete—in Lacanian terms, that there is a “lack in the symbolic Other.” Thus, society exists as a totality only insofar as the social subject *posits* its existence as such through the mediation of empty signifiers (2001, p. 197).

It is in the operation of master-signifiers, continues Glynos, that epistemological incapacity is transformed into a positive ontological condition. We use such master-signifiers every day, without a second thought (“America,” “society,” “men,” “history,” etc.). Such signifiers, should, by virtue of their vagueness, their breadth of abstraction, surrender to a type of amorphous meaninglessness. Indeed, one might expect them to buckle under the weight of the signifying load heaped upon them. Yet this does not happen.

What seems to be the impossibility of master-signifiers, the fact that no one totalizing or exhaustive definition can be allocated to them proves also to be their condition of possibility. Put differently: a prospective master-signifier needs to be over-stretched, burdened with the task of “incommensurable universal signification” before it comes to operate as such. It is only by being over-loaded in this way that a signifier begins to exceed the work of mere signification and becomes “super-charged,” self-referential. Hence the initially puzzling idea that a master-signifier does not in fact *signify anything at all*, beyond itself.

A master-signifier is in this sense akin to a recursive loop in the functioning of signification itself. The signifying impulse gets caught in a self-affirming pattern, in a repetitive circuit, hence the circular, self-instantiating grounds of such “transcendent” terms. It is this endless self-referral of master-signifiers which means that they come, paradoxically, to “count” more than the surrounding signifiers upon which they operate.Lacanian theory thus both extends and yet departs from Structuralism. As Dolar (1999) explains:

[A]ny notion of structure, far from being simply differential, a balanced matrix of permutations (as in Levi-Strauss) necessarily gives rise to a ‘Master-signifier’, a structural function that power gets hold of, but which is in itself empty, a pure positivisation of a void (p. 87)

### …Social fantasy

If we appear to have drifted too far toward theoretical abstraction, too far away from our case study of Mandela as master-signifier, then consider Nuttall's (2013) commentary on the mortality of the former South African president:

With the fact of his late old age comes the sense that [Mandela] marks a deep void at the heart of a place that has always struggled to mask what it feels might be an emptiness at its center, that has struggled to define itself as a nation and to draw together its many fragments into a sustained sense of commonality, in the wake of a long racist past. We approach alongside him the anxiety or anguish that South Africa is neither a concept nor an idea—just a physical place, a geographical accident (Nuttall, 2013).

Mandela, thus approached, is the signifier that covers the void of meaning that is South Africa. One appreciates how, in late 2013, Mandela's immanent death may have been thought to represent the end of the fantasy, the point at which the concept of South Africa ceased to work as anything other than an imaginary construct. Perhaps it is the case—easy enough to imagine if Mandela's legacy were erased from history—that, following Nuttall (2013) “South Africa” is no more than the name for a set of historical contingencies to which no historical essence, no grand march of progress can rightly be said to apply.

The above reference to fantasy, to the *mythical* dimension of Mandela as master-signifier, is apt. As Stavrakakis (1999) observes, the symbolic construct articulated via a master-signifier “can function properly only within a certain fantasmatic frame; the empty signifier can function only as an *object petit a*” (p. 81), that is, in relation to the object-cause of desire which holds a given fantasmatic scenario in place. A master-signifier, in other words, is never merely objective in its meaning and value, but is animated rather by belief, by the imagination, the libidinal and fantasmatic aspirations of those who have invested in it. There is thus some truth in political evaluations that suggest that Mandela's greatness “is mainly a creation of the collective imagination” (Beresford, 2004). We might rephrase this idea psychoanalytically simply by noting that Mandela—and master-signifiers more generally—are targets of intense transference reactions. In speaking of Mandela as master-signifier, we convey not only that Mandela has become a focal-point of multiple subjective investments, but the effect of a shared fantasy underwriting a given view of social reality.

### …and retroactive principle of articulation

We would be remiss if were not to mention the *retroactive* dimension of master-signifiers. That there are cardinal points in discourse, areas of marked libidinal intensity, is always already the result of master-signifiers ordering the field of signification and affect alike. This is particularly salient in the case of Mandela, where—in line with the narrative role typically accorded the former president—it is easy to fall prey to the illusion that Mandela was always destined to assume the role of the unifying, transcendent signifier of the post-apartheid nation. Contrary to such assumptions we should insist: there was nothing pre-destined about the success of Mandela as pre-eminent master-signifier of South Africa's “long walk to freedom” (Mandela, 1994). The retroactive effect of historical order—indeed, of narrative cohesion—is precisely the result of a master-signifier knitting together the disarray of contingent signifiers and events and producing a sense of cogency and inevitability. This helps us make a further general point: as components of ideology, master-signifiers don't so much eliminate opposing terms as *re-articulate* them. Rival ideological terms come thus to be re-integrated, put into a different set of signifying relations. This is a situation in which nothing can be said to have changed (in the sense of new content being added to the discourse) yet where everything is, nevertheless, utterly transformed (there has been a reshuffling of discursive elements under the ascendency of a new master-signifier).

Such a reshuffling of discursive elements is apparent in the changing historical role of Mandela as master-signifier. What had for many years been a master-signifier within apartheid ideology, a signifier predominantly associated with a variety of others—“communist threat,” “anti-white,” “violence,” “terrorist”—eventually came to operate as the compositional center, the focal-point of the discourse of the new South Africa. It should be clear then that the role of the master-signifier in arranging a discursive field is anything but a once-and-for-all operation. It is an instance rather of how signifiers, which still play a role in other discourses, come to be rearranged, subjected to a new constellation.

## The master-signifier in psychical economy

We stressed above the need to grasp the role of master-signifiers not only as elements within the functioning of political discourse, but as components in the psychic economy of subjects. A master-signifier, after all—as we have seen—is fueled by an object *a*, and animated by subjective belief, by the fantasies and desire of those who have invested in it. This theoretical context, of how master-signifiers operate at the level of psychical economy, is, we would argue, irreducible in an adequate understanding of the concept. A master-signifier's strength, in this respect, is not merely linguistic—or so we might argue—but also *affective* in nature, a question of passionate attachments, of the libidinal ties and subjective identifications that it gives rise to. We might put it this way: the operation of master-signifiers itself entails a type of libidinal economy inasmuch as it involves a distribution and arrangement of affects.

Here though we must proceed cautiously. From a Lacanian perspective, linguistic operations are the very stuff of psychical functioning. So, (mass) identification, as we have intimated above, is as much a symbolic and political as it is a psychical and *subjective* process; it is as much about the unconscious investments of individual subjects as about the consolidation of hegemonic forms of discourse. The repeated Lacanian injunction to attend to the signifier, to the symbolic, indeed, to *the trans-subjective*, means that social and individual are never easily separated. In much the same way, the question of affective bonds should not be seen as extrinsic to the role of signification. “Affect is not something external, added to the symbolic, but an internal component of it” (Laclau, in Glynos and Stavrakakis, 2010, p. 235). This is to say that the affective charge of a given signifier or representation does not exist *outside of the realm of signification*. Affects should be grasped as the outcome of relations between signifiers. Laclau continues:

Affect is not some vague emotion external to signification, for it can only constitute itself on the basis of overdetermining a signifying chain…the signifier ‘rat’ [in Freud's case of the Raman] is so affectively overcharged because it evokes—overdetermines—a plurality of currents of unconscious thoughts—money, sex, the father, and so on (Laclau, in Glynos and Stavrakakis, 2010, p. 235).

Affect must thus be approached as an *internal* component of signification, rather than understood as something added to the operations of signification. Any rigid demarcation between affect and representation, signifier and *jouissance*, must thus be brought into question: “the distinction between affect (cathectic investment) and the symbolic is…intra- and not extra-discursive” (Laclau, in Glynos and Stavrakakis, 2010, p. 239). In Lacan's discourse theory the relation between the master signifier (S1), the other signifiers (S2) and the object *a* expresses the internal nature of this affective relationship. Signifying articulation itself (S1 → S2) creates the insistence of a libidinal element (*a*) at the level of the not-said, which further gives rise to positing a master-signifier. It is helpful now to switch to a clinical focus on the master-signifier in relation to the subject; doing so brings to light aspects of the concept that may—and typically do—remain otherwise neglected.

## S1, S2, and the divided subject

Strictly speaking, it makes no sense to speak of a “master-signifier” in a way that implies it can be disengaged from the multitude of other signifiers of which it is a part. Lacan's quasi-mathematical notion of the 1960's emphasizes as much: a master-signifier (S1) is always itself contingent on a dispersed array of elements (S2) that come, retroactively, to operate as a bounded field of knowledge. Hence Fink's (1995) description of the master-signifier as “the nonsensical signifier” which is “brought into the movement of language…“dialecticized” through the action of various S2s” (p. 75). Indeed, logically S1 gives rise to the articulation of S2, but in terms of the signification process S2 *retroactively* determines S1.

Crucially however, not only does the passage from S2 to S1 render a disorganized set of signifiers legible, it also arranges a set of otherwise disparate signifiers into *practicable* forms of knowledge (*savoir faire*). Such a process, crucially, implies a subject, precisely the subject for whom the relation of knowing thus produced becomes operable (the subject “that represents a signifier for another signifier”). S1 ← S2 is thus a type of Lacanian shorthand for *subjectification*. This is axiomatic for Lacan: the functioning of the signifier “entails” the subject, which, in his view, is precisely an effect of references between signifiers. Just as S1 cannot be separated from S2—it wouldn't be a master-signifier if this were the case—so the S1 ← S2 relationship, as an instance of signification, cannot be divorced from the subject that it necessarily involves. Given the use of the signifier, an enunciating position starting from which signifying articulation takes place needs to be presumed. However, and crucially, this position is never fully or adequately represented by any of the signifiers that are thus articulated (Feyaerts and Vanheule, 2015). To be sure: the S1 ← S2 relationship necessarily entails a subject, not a whole or integrated subject however, but a subject split by the incommensurability of the signifying process itself. In terms of Lacan's discourse of the master (Figure 1), this enunciating subject is the *$* that is concomitant with the articulation of S2 and S1, which is what the upward (vertical and diagonal) arrows starting from *$* express. Indeed, Lacan makes a crucial distinction between the *enunciating* subject (*$*)—that is, the speaking subject—and the *enunciated* subject, the subject that is spoken, produced via the articulations of the signifying chain (S1 → S2).

Let us risk an elementary example which may bring this idea to life. When an individual occupies a role in relation to someone else—say that of fatherhood in relation to a child—a set of elementary signifiers is distributed. A master-signifier (“father”) is assumed, and starting from this S1 an array of culturally related signifiers is evoked (taking care, guiding the child, relating to the mother…). When in daily life interactions take shape, an enunciated subject gets created (“Daddy says no; you have to listen to me!”). Yet, typically people never identify completely with the master-signifiers they assume. Next to being a father the same individual may also identify as a “lover” in relation to a partner, or as a “hooligan” in relation to friends; signifiers that are not always compatible with the identification with fatherhood. Moreover, concrete actions taken by the father don't make up a whole (one time he helps, another moment he screams at the child). Nonetheless all these actions emanate from the same person, which is what the concept of the divided enunciating subject makes clear.

Indeed, the retroactive S1 ← S2 relationship is not to be understood as of an exclusively intellectual sort, or as limited to the field of purely linguistic signification. This relationship needs rather to be grasped as pertaining to signification in a far more encompassing sense. Signification here includes not just instances of propositional knowledge, but types of expertise or practical “know how” (as in the distinction between *connaissance* and *savoir*, respectively). The point is worth reiterating: for Lacan, knowledge (S2) in its relation to a master-signifier (S1) is meant also in the sense of *savoir faire*. Importantly, both such forms of knowing rely on the rudimentary differentiation between signifiers—that is, a minimal differential element—required by all forms of knowing. We might consider a basic example: a hungry infant fumbling at the mother's breast, trying to find the nipple. There is a rudimentary sort of signification (or comprehension) occurring here; in differentiating the nipple from other parts of the breast the infant “knows” something: how to go about feeding. S1 ← S2 can thus be said to extend to a wide range of ostensibly non-linguistic or practical forms of knowing-how beyond the realm of propositional knowledge. In both such cases the knowing in question can be conceived as precisely *a relation between signifiers*.

## “Dialecticizing” the master-signifier

From a clinical perspective what matters is not simply to spot a master-signifier in the speech of a patient—that is easy enough—but *to query the particular role* this signifier plays for them. We need ask: what task is being performed by this signifier, and, more pointedly, what is being elided, or repressed—by the S1 ← S2 relation? The clinical literature makes this point of emphasis quite clear. “[M]aster signifiers,” says Bailly (2009), “have become quite detached from their signifieds” so as to

carry out the function of changing the meaning of the signifying chain into one that supports the ego. It is one of the main tasks of analysis to…bring to light the side of them [master-signifiers] hidden in the unconscious” (p. 64).

“[I]n the analytic situation” says Fink (1995), “a master-signifier presents itself as a dead end, a stopping point.[it is] a term, a word, or phrase that puts an end to association, that grinds the patient's discourse to a halt” (p. 135). The contrast between examples drawn from the political literature and the clinic is here apparent: in the former, master-signifiers represent an apparent over-flow of meaning; in the clinic they are typically the point where signification stalls, where meaning closes down. One of the chief goals of analysis is thus to “dialectize” master-signifiers which freeze the enunciating subject; the objective is to clear the blockage, to shift the relation of domination imposed by S1s. It is in line with this conceptualization that Fink refers to the master-signifier as “subjugating the subject” (p. 78), as “the signifier that commands or [acts as] commandment” (p. 135). It is by bringing master-signifiers into relation with other signifiers, signifiers that may shift the given locked (S1 → S2) relation, by turning “dead ends…into through streets” (p. 78), that one succeeds in “dialectizing” the master-signifier. One likewise aims at “dialectizing” the enunciated subject that gets articulated along the lines of the S1. Returning to our discussion of Mandela, we might ask: what other narratives of the emergence of the post-apartheid nation might be possible; what other accounts might take us beyond the horizon of Mandela-associated meanings? More revealing yet, perhaps: why the need to subscribe to the Mandela myth, and what might this myth conceal?

We need to pay careful attention to Fink's terminology. The fundamental structure of signification, as we have seen, entails the establishment of a link between a master-signifier and other signifiers, a link furthermore which ensures that—Fink (1995) uses a neutral term here—*subjectification* takes place. By linking S1 to S2, *$* gets created. When the master-signifier is isolated, difficult to dialectize, when a S1 → S2 relationship is locked, “it *subjugates* the subject”: the enunciated subject then covers and hides the evanescent enunciating subject. Yet, when it is linked up with a new or disruptive set of signifiers, *subjectivization* results, and an enunciating subject that does not coincide with its previous utterances comes to the fore. A successful analysis could be said to pivot on the difference between these two modes of the subject: on the movement from subjugation to subjectivization. To recapitulate:

[In analysis] one tries to introduce an outside…of this S1…If we can bring this S1 into some other kind of relation with [surrounding signifiers]…then its status as a master-signifier subjugating the subject changes. A bridge is built between it and another linguistic element…the analysand is no longer stuck at that particular point of his or her associations…A meaning of the master-signifier is created…the subject is once again split…having come to be momentarily in the forging of a link between S1 and S2 (Fink, p. 78).

It is the creation of a new relationship between a given S1 and other signifying elements “which allows for a subjective *position*” (Fink, p. 78). What is crucial to grasp here is that this subject does not transcend their status as the split subject of the unconscious. The subject cannot bypass or supersede the division which, after all, is, for psychoanalysis, constitutive of the subject as such (hence Fink's reference to the subject being “once again split” (p. 78) through the act of enunciation). If anything, the split is realized in a more pronounced way. Yet this is precisely what psychoanalysis is all about, peering deeper into, confronting, the constitutive split that *is* the subject, and considering alternative—less subjugated yet nevertheless divided—forms of subjectivization that become possible in the process.

## The unenunciated aspect

Master-signifiers then do not only anchor meanings and fix the nodal-points of a given discourse; they also *structurally repress* other signified meanings through, for example, the subject's insistence that “‘this is the way things are,’ that it is not subject to challenge or dissent” (Parker, 2005, p. 170). The very process of discursive insistence—be it in the endless reiteration of the master-signifier or in the self-referring circularity upon which it depends—effectively shuts down differing interpretations and dissent (Parker, 2005).

While, for Bailly (2009) master-signifiers are the very backbone of the human subject, “they are also, perhaps in negative form (in the sense of the negative of a photograph), the stuff of denegation” (p. 61). Master-signifiers, furthermore, “usually mask their opposites…they exist in a polarized form” (p. 63). The openly expressed aspect of the master-signifier props up an ego—that is, the imagined identity of a subject or community—while the enunciating or *un*enunciated aspect remains “buried in the unconscious…constantly pushing up its opposite number” (p. 63). The function of the master-signifier is thus to redirect potentially painful or anxiety-provoking signifiers, and to do so in such a way “that a signifying chain with the opposite, bearable, or even comforting meaning emerges” (p. 63). The critical injunction suggested by Bailly's comments is vital, namely the idea that we investigate the negated opposite or underside of a given master-signifier, that we ask ourselves what the master-signifier holds at bay and keeps beyond the domain of the thinkable?

How then to understand this unennuciated aspect in respect of the massive proliferation of commemorative practices that took place in South Africa around the time of Mandela's death? The intuitive response would be to say that these significations celebrated Mandela's life, affirmed all that he had achieved. Then again, following the insight that master-signifiers often mask their opposites, we might adopt a different hypothesis. Contrary to assuming that the endless profusion of Mandela signifiers speaks to the historical objectivity of the Mandela legacy, it is worth wondering whether this activity is fueled rather by a fantasy, *a need to believe*. The symbolic density connoted by such activities and representations is a clear signal that a society is fortifying a mode of belief, concretizing a cherished set of ideals and subjective/societal investments. In short: we don't erect monuments simply to celebrate and affirm what we already know; we build and sustain monuments so that we will continue to know and believe what may otherwise be erased through time and through various forms of uncertainty or doubt.

We might question then whether this commemorative impulse was propelled by the immanent failure of, or disbelief in, the vision of an integrated South Africa that Mandela championed, and, furthermore, whether this multitude of symbolic gestures attempted—desperately perhaps—to affirm such a unified social reality, despite the mounting evidence of growing social and political division. The surge of commemorative practices perhaps indicated then less the absolute truth of the political changes Mandela helped bring about, than the fact that without the constant activity of Mandela signification, South Africans feared they *might fail to believe* in such changes—promised or otherwise—and begin to fear that many of the country's old divisions might resurface.

This speculative exercise sheds some light on the role of the master-signifier in bolstering an ego-affirming fantasy and concealing far less consoling signifiers. We can extend this by asking how Mandela operates also as a site of repression. Posel's (2014) recent discussion of the politics of spectacle in post-apartheid South Africa proves an invaluable resource here. A crucial part of Posel's analysis concerns the controversial figure of Julius Malema, the “angry, unruly bad boy of post-apartheid politics” (p. 32) who Posel positions as a type of “negative Mandela”:

Malema entered the public sphere as a counterpoint to Nelson Mandela—unsettling the iconography of non-racialism, reasserting an angry and confrontational version of race that reinstated the specter of violent conflagration that Mandela's ‘miracle’ held at bay (p. 32).

The dynamic between the portrayals of these men has a clear historical dimension inasmuch as it invokes an unprocessed past, and the theme of repression, although not described in overtly psychoanalytic language, is clearly present:

juxtaposing the spectacular public life of Julius Malema…with that of Nelson Mandela draws attention to…the ‘haunting’ presence of the past…If the mythic Mandela championed the project of ‘national reconciliation’—his symbolic powers put to the work of performing ‘non-racialism’—Malema emerged as the symbolic counterpoint, marking the limits of this project: a ghostly reminder of the abiding racial wounds that have endured (p. 35).

So while it is true that the mythic Mandela is both “the condition and counterpoint of Malema's public persona” (p. 35), Posel's further descriptions suggests also a type of negation, even—as in the above references to the haunting presence of the past—a return of the repressed:

If Mandela was the national archetype of adult wisdom, the Black man willing to reconcile and embrace fellow White citizens, Malema styled himself as the quintessentially angry Black man: youthful militant refusing to cow-tow to his political elders, masculinist “revolutionary,” avowedly confrontational on racial issues (p. 39).

While an apt characterization, “symbolic counterpart” does not quite do justice to the dynamic underlying the relationship between these signifiers. Malema, we might venture, is the repressed truth of Mandela^3^. Malema emerges as the underside, the return of what was so effectively repressed by Mandela^4^. The remarkable success of Mandela as master-signifier may be said to have much to do—particularly for white South Africa—with what it kept at bay, namely, all that today is signified by the name Malema. In this sense, it is not only—to invert Posel's argument—that Mandela be seen as a condition of possibility for Malema, but Malema, or what Malema signifies, that acts as *a condition of possibility for Mandela*.

## Conclusion

As we hope is by now apparent, our objective here has not been to discredit political and sociological applications of the master-signifier concept. This literature usefully stresses how master-signifiers operate ideologically at the level of discourse to engender effects of hegemony, to knit together a field of discursive elements, and thus to consolidate forms of mass identification. In all of these ways, we stand to benefit from this literature. That being said, this literature does sometimes run the risk of equating the master-signifier with the processes of discursive hegemony in ways which overlook—or underplay—the role of unconscious dynamics of repression^5^.

Reference to the clinical literature shows how the master-signifier operates also as a psychical—and not merely discursive—function, and does so at the level of the subject, in accordance with a libidinal economy, to defensively bolster an ego, and to mobilize effects of fantasy and repression. Any adequate analysis of a master-signifier needs to consider not only apparent overflows of meaning, but also those points where meaning seizes up and associations are halted; not only how multiple meanings come to be articulated under the ascendency of a hegemonic signifier, but the various repressed and negated signifiers—the unenunciated and unarticulated—that the master-signifier *as ego-function* routinely elides^6^. Simply put: the master-signifier is not a properly psychoanalytic concept if it fails to take into account the role of desire, which is inevitably also to consider the unconscious and the multiple defenses that arise around it.

### Conflict of interest statement

The authors declare that the research was conducted in the absence of any commercial or financial relationships that could be construed as a potential conflict of interest.

## Footnotes

^1^While the object *a* can be situated at the level of the other, it should not be seen as an element that attunes subject and other. On the contrary, what makes the neurotic situate the object *a* at the level of the other, is a deep uncertainty as to what the other wants. The absence of a harmonious link provokes the question of the other's desire, elicited, for example, by the gaze of he who looks at me, or the silence of the one listening to what I say.

^2^We include counter-identification as itself a form of identification here, such that even rejections of the historical figure or master signifier in question may be said to maintain a tact (indeed, negative) identification with what is being rejected.

^3^There is a nice formal bridge here at the level of the signifier, which will not go unnoticed by Lacanians: Mandela and “Malema” are near homonyms.

^4^Consider the historical context provided by Posel; surveys in the 1990's: showed rising levels of White fear at the prospect of an escalating violent conflagration—reinstating the longstanding specter of the angry Black mob…the dreaded prospect of Black insurrection against White minority rule that had dominated the White collective imagination since apartheid's inception (2014, p. 49).

^5^It is not difficult to understand why this is so: discourse analysis, in adherence with the later Foucault's (1990) emphasis on the *productions* as opposed to *suppressions* of discourse, has long since proved averse to the concept of repression, seeing it as a concept freighted with problematic “psychological” (and psychoanalytic) presumptions. Lacan approaches the issue differently: the concepts of repression and the unconscious are to be retained, but theorized precisely via the operations of the signifier.

^6^True enough, the psychoanalytic dimension of the concept is not neglected by all political theorists. Stavrakakis (1997) and Glynos (2001), to note just two examples, remain attentive to the dynamics of repression and the fantasy-inducing aspects of master signifiers, aspects notably absent, as noted above, in definitions such as that provided by Gunkel (2014).
